# Laparo-endoscopic management of chole-choledocholithiasis: Rendezvous or intraoperative ERCP? A single tertiary care center experience

**DOI:** 10.3389/fsurg.2022.938962

**Published:** 2022-08-31

**Authors:** Elpiniki Lagouvardou, Gennaro Martines, Giovanni Tomasicchio, Rita Laforgia, Angela Pezzolla, Onofrio Caputi Iambrenghi

**Affiliations:** ^1^ Azienda Ospedaliero Universitaria Consorziale Policlinico di Bari, Bari, Italy; ^2^ Department of Emergency and Organ Transplantation, School of Medicine, University of Bari Aldo Moro, Bari, Italy

**Keywords:** chole-choledocholithiasis, single-stage approach, ERCP, rendezvous, outcomes

## Abstract

**Introduction:**

The management of chole-choledocholithiasis remains a matter of debate to preserve minimal invasive management and different options have been proposed, with single- or two-stage approaches. Two techniques of single-stage approach are intraoperative ERCP and laparoscopic rendezvous, which have the great advantage of reducing the length of hospital stay with increased patient compliance. This retrospective study aims to evaluate and compare the efficacy and safety of intraoperative ERCP and rendezvous technique for more than 15 years.

**Materials and methods:**

Clinical records of 113 patients who underwent single-stage management for chole-choledocholithiasis between January 2003 and December 2020 were retrospectively reviewed using a prospectively maintained database. Patients were separated into two groups: those managed with intraoperative ERCP and those with rendezvous, and their intraoperative and postoperative parameters were compared. All patients were followed up for 6 months in an outpatient setting.

**Results:**

A total of 68 (60%) patients were treated with intraoperative ERCP, while the remaining 45 (40%) were treated with rendezvous. There were no significant differences in terms of comorbidities. ERCP was performed with a median operative time of 145 min (104–168) and an endoscopic time of 27 min (15–36). Meanwhile, rendezvous was performed with a significantly lower operative [120 min (94–147)] and endoscopic time [15 min (12–22)]. No intraoperative complications were recorded. Patients treated with rendezvous had a significantly shorter median hospitality stay [4 (3–5) vs. 3 (2–4) days, *p* < 0.05]. No hospital readmissions or mortalities were observed in either group after 30 days. Ten mild pancreatitis cases were observed, mainly in the intraoperative ERCP group (9 vs. 1, *p* < 0.05), and all were treated conservatively. Only two patients treated with intraoperative ERCP developed biliary complications later on.

**Conclusion:**

Laparoscopic rendezvous should be considered a preferable alternative to intraoperative ERCP for the treatment of patients with concomitant CBD stones and gallstones.

## Introduction

Choledocholithiasis has been reported in 10%–15% of patients undergoing cholecystectomy for gallstones (1). While laparoscopic cholecystectomy (LC) has been established as the gold standard for symptomatic gallstones, the management of chole-choledocholithiasis remains a matter of debate. Before the era of LC, open cholecystectomy with common bile duct (CBD) exploration was the standard treatment for these patients. Nowadays, to preserve minimal invasive management, different options have been proposed, with single- or two-stage approaches (2).

In daily clinical practice, the two-stage management, consisting of preoperative or postoperative endoscopic retrograde cholangiopancreatography (ERCP) with sphincterotomy and CBD clearance followed by LC, is most frequently used (2, 3). The European Association for the Study of the Liver (EASL) recommends delaying surgical therapy for several days after endoscopic intervention to assess the possible development of post-ERCP pancreatitis, which occurs in 10% of cases (4, 5). This two-stage strategy increases length of hospital stay, increases the costs, and requires two different anesthesiologic sessions (6).

To date, various techniques have been proposed to treat choledocholithiasis in a single laparoscopic session, including LC plus laparoscopic CBD exploration, LC plus trans-cystic laparoscopic bile-duct clearance, LC plus intraoperative ERCP, and LC plus rendezvous (7, 8). Laparoscopic CBD exploration and trans-cystic bile duct clearance has not gained popularity among the surgical community. These techniques have the advantage of preserving the function of the sphincter of Oddi, while also reducing the overall hospital stay and costs (9). However, these techniques require strong clinical experience, a long learning curve, and advanced laparoscopic skills, especially when a T-tube is used (2, 3).

Intraoperative ERCP and rendezvous techniques have the great advantage of reducing the period of hospital stay, while increasing patient compliance, without the disadvantages of laparoscopic CBD exploration and trans-cystic bile duct clearance.

Several meta-analyses have compared single-stage with two-stage management without a reliable conclusion, considering that the two therapeutic strategies are equally safe and feasible for the management of concomitant CBD stones and gallstones (9, 10). However, few studies compare intraoperative ERCP versus rendezvous for the treatment in one-stage management of chole-choledocholithiasis.

This retrospective study aims to evaluate and compare the efficacy and safety of intraoperative ERCP and rendezvous technique plus LC in the treatment of patients with concomitant CBD stones and gallstones for a period of more than 15 years.

## Materials and methods

A retrospective observational study was carried out using a prospectively maintained database of patients with concomitant gallstone and CBD stones in a tertiary surgical-endoscopic unit in the period between January 2003 and December 2020. Patients over 18 years old, with symptomatic gallstones, a preoperative diagnosis of CBD stones, and those who completed a 6-month follow-up were included in the study. Exclusion criteria were the presence of intrahepatic biliary stones, malignant pancreatic or biliary tumors, suppurative cholangitis, asymptomatic gallstone, contraindications to ERCP, and pregnancy. Preoperative diagnosis of concomitant chole-choledocholithiasis was routinely performed based on clinical presentation, blood panels, abdominal ultrasonography, abdominal CT scan, and/or magnetic resonance cholangiography. All patients underwent an intraoperative cholangiography to confirm the presence of CBD stones. Patients were separated into two groups: in the first group, CBD stones were managed with intraoperative ERCP, and in the second group, with rendezvous. All procedures were performed by the same endoscopist over the same period of time, and LC was performed by different surgical teams. Demographic data were collected including age, gender, ASA Physical Status Classification System, and comorbidity. The operative time and open conversion rate were parameters collected intraoperatively, and the operative time included the time calling the endoscopist and the time required to set up the endoscopist equipment. Postoperative parameters registered included the length of hospital stay and the incidence of hospital readmission within 30 days. Postoperative complications were recorded in accordance with the published Clavien–Dindo classification (11). All patients were followed up for 6 months in an outpatient setting.

### Intraoperative ERCP and rendezvous procedure details

All patients provided informed consent after a thorough explanation and counseling of the benefit–risk ratio. Under general anesthesia, a 4-trocar technique was used for LC. During LC, a 5F catheter was inserted through an incision in the cystic duct for intraoperative cholangiography. After the cholangiogram, the patients enrolled in the first group underwent intraoperative ERCP. The first jejunal loop was identified and clamped to avoid bowel insufflation during the endoscopic procedure. The duodenoscope was inserted into the descending duodenum. After cannulation of the papilla of Vater, a guidewire was placed. Endoscopic sphincterotomy was performed, and CBD stones were removed using a Dormia^®^ basket. In patients with preoperative or intraoperative diagnosis of difficult cannulation, and those belonging to the second group, the rendezvous technique was performed. The difficult biliary cannulation was defined by the presence of one or more of the following: more than five contacts with the papilla while attempting to cannulate; more than 5 min spent attempting to cannulate following visualization of the papilla; more than one unintended pancreatic duct cannulation or opacification, as suggested by the ESGE (12).

A 5-Fr catheter was inserted in the cystic duct under direct laparoscopic vision. A guidewire was advanced through the catheter into the duodenum, and the catheter was clipped to the cystic duct. Duodenoscopy was then performed with a duodenoscope. The guidewire was advanced into the sphincter of Oddi and duodenum and pulled out of the patient's mouth. The papilla was directly cannulated, and sphincterotomy was then performed. A Dormia^®^ basket was introduced to clean the biliary tree at the end of sphincterotomy. The endoscopic procedure ended with the removal of guidewire and endoscope through the patient's mouth. The laparoscopic procedure was completed after clipping the cystic duct and by dissecting the gallbladder from the liver bed. Drainage was installed when appropriate.

### Statistical analysis

Continuous parameters were reported as median and interquartile ranges. Categorical variables were recorded as numbers and percentages where appropriate. Comparisons of categorical variables were performed by the *χ*^2^ and Fisher's exact test where appropriate. Comparisons between groups were made using the Mann–Whitney *U* test. A *p-*value <0.05 was considered statistically significant. Statistical analysis was carried out using RStudio (R version 4.0.3 10/10/2020 Copyright© 2020, The R Foundation for Statistical Computing).

## Results

A total of 113 patients affected by chole-choledocholithiasis were treated with one-stage management between January 2003 and December 2020. Sixty-eight of them (60%) underwent intraoperative-ERCP plus LC [median age 68 (62–74), 45% males], and the remaining 45 (40%) underwent rendezvous plus LC [median age 59 (53–66), 51% males]. Both were included in the study according to the study selection criteria. There were no significant differences in terms of comorbidities such as cardiovascular disease, diabetes mellitus, and chronic obstructive pulmonary disease between the two groups. None of the patients had an ASA preoperative score of IV, but most of them (51.4% vs. 46.7%) had mild systemic disease (ASA II), with no significant differences between the two groups. The relevant preoperative characteristics of included patients are shown in Table 1. LC plus intraoperative ERCP was successfully performed with a median operative time of 145 min (104–168) with an endoscopic time of 27 min (15–36). Meanwhile, rendezvous plus LC was performed with a significantly shorter operative time [120 min (94–147)] and endoscopic time [15 min (12–22)] when compared with intraoperative ERCP group cases (Table 2). No intraoperative complications were recorded. The laparoscopic-endoscopic procedure was successfully performed in all patients, except in one case of the intraoperative-ERCP group, where it was not possible to complete the procedure laparoscopically and thus was converted to open surgery due to the great difficulty of direct cannulation of the biliary tree for the presence of a duodenal diverticulum. Patients treated with rendezvous had a significantly shorter median hospitality stay when compared with intraoperative ERCP group [4 (3–5) vs. 3 (2–4) days, *p* < 0.05]. No hospital readmissions or mortalities were observed in either group after 30 days. The overall morbidity and specific complications of the two groups are reported in Table 3. Ten mild pancreatitis cases were observed, mainly in the intraoperative ERCP group (9 vs. 1, *p* < 0.05), all treated conservatively with fasting, proton pump inhibitors, antibiotics, and somatostatin management. Bleeding occurred in three cases in the intraoperative ERCP group, requiring endoscopic hemostasis in one case, whereas the remaining was treated conservatively. One patient developed postoperative subhepatic abscess requiring CT-guided percutaneous drainage. All patients were followed up for 6 months in an outpatient setting. Only two patients developed later biliary complications: one developed CBD recurrent stones, and another patient had papillary stenosis both treated with ERCP.

**Table 1:** Relationship between demographic features and comorbidities of intraoperative ERCP and rendezvous groups.

Demographic data			
	Intraoperative ERCP *n* = 68	Rendezvous *n* = 45	*p*-value
Age	68	59	0.65
(Years)	(62–74)	(53–66)	
Gender (M/F)	31/37	23/22	0.83
ASA			
– I	11 (16.2%)	9 (20%)	0.78
– II	35 (51.4%)	21 (46.7%)	0.75
– III	22 (32.4%)	15 (33.3%)	1
– IV	0	0	–
Comorbidity			
– Cardiovascular	30 (44.2%)	20 (44.4%)	1
– Diabetes	16 (23.6%)	9 (20%)	0.83
– Respiratory	11 (16.2%)	7 (15.6%)	1

ERCP, endoscopic retrograde cholangiopancreatography.

**Table 2:** Relationship between intraoperative data of intraoperative ERCP and rendezvous groups.

Intraoperative data			
	Intraoperative ERCP *n* = 68	Rendezvous *n* = 45	*p*-value
Operative time			
– Total time (min)	145 (104–168)	120 (94–147)	*<0.05
– Endoscopic time (min)	27 (15–36)	15 (12–22)	*<0.05

*statistically significant.

ERCP, endoscopic retrograde cholangiopancreatography.

**Table 3:** Overall morbidity and specific complications of intraoperative ERCP and rendezvous groups.

Postoperative data			
	Intraoperative ERCP *n* = 68	Rendezvous *n* = 45	*p*-value
Hospital stay	4 (3–5)	3 (2–4)	<0.05*
Readmission <30 days	0	0	—
Complications			
– Bleeding	3 (4.2%)	0	0.27
– Pancreatitis	9 (13%)	1 (2.2%)	<0.05*
– Bile leak	0	1 (2.2%)	0.3
– Cholangitis	1 (1.4%)	0	1
– Abscess	1 (1.4%)	0	1
Clavein–Dindo			
– 0	53 (78%)	43 (95.5%)	<0.05*
– I	9 (13%)	2 (4.5%)	0.19
– II	4 (6%)	0	0.14
– IIIa	2 (3%)	0	0.5
– IIIb	0	0	—
Later biliary complications			
– Biliary/papillary stenosis	1 (1.4%)	0	1
– Recurrence CBD stones	1 (1.4%)	0	1

*statistically significant.

CBD, common bile duct; ERCP, endoscopic retrograde cholangiopancreatography.

## Discussion

Management of simultaneous gallbladder and CBD stones has improved since the advent of laparoscopic surgery but remains a challenging surgical problem. To date, there are no guidelines recommending the optimal timing and treatment for chole-choledocholithiasis. The two-stage management is currently preferred by many hospitals, but several disadvantages arise with this approach, including increased hospital stay, increased costs, and decreased patient compliance (3, 13, 14). The timing between the two procedures ranges from 24 to 72 h up to 6 weeks, and this delay could lead to a 10% risk of CBD stones recurrence (15). Recent meta-analyses comparing the single-stage and two-stage management showed equivalent clinical complications and outcomes, with better cost-effectiveness and reduced length of stay in the single approach (3, 16, 17). In this regard, different single-stage techniques have been proposed, and most surgeons and gastroenterologists are well-versed in intraoperative ERCP plus LC and rendezvous plus LC (13). However, intraoperative ERCP has several limitations, mainly created by the need to perform ERCP with the patient in a supine position, which makes the retrograde cannulation of the papilla more intricate (18). Moreover, the need for endoluminal insufflation for endoscopic vision could interfere with LC due to the distension of the small bowel. For this reason, in our clinical practice, we positioned an atraumatic laparoscopic clamp on the first jejunal loop to reduce intestinal distension. Hence, intraoperative ERCP is associated with failure to cannulate the ampulla of Vater ranging from 4% to 18% (2, 19), with the risk of postoperative pancreatitis due to inadvertent pancreatic duct cannulation. In rendezvous, where the guidewire is inserted through the cystic duct, the identification and cannulation of the papilla are facilitated, minimizing the risk of inadvertent pancreatic duct cannulation and subsequent risk of pancreatitis (2, 18). In our experience, rendezvous has a higher success rate of CBD cannulation compared with intraoperative ERCP (2). In just one (2.2%) patient, it was not possible to cannulate the CBD in the presence of a duodenal diverticulum, and in this case, conversion to open surgery was necessary. In our cohort of patients, in line with the literature, only 1 (2.2%) patient reported pancreatitis after rendezvous, while it occurred in 9 (13%) patients after intraoperative ERCP, and all were treated with conservative management. The safety of this procedure is reinforced by literature that underlines a statistically significant lower serum amylase levels after rendezvous when compared with traditional ERCP (20, 21).

In the literature, the single-stage approach is a cost-effective treatment for decreased hospital stay and lower rate of late biliary complications (22). Our results are in line with literature, showing a shorter hospital stay, with only 1–2 more days with respect to the traditional hospital stay for LC for uncomplicated biliary colic (23). Furthermore, the rendezvous cohort had a significantly shorter hospital stay when compared with the intraoperative ERCP cohort [3 (2–4) vs*.* 4 (3–5), respectively] with more than 95% of patients without postoperative complications and with a grade 0 of Clavein–Dindo classification. Both procedures had significantly shorter hospitality stay than the average 5–6 days reported for two-step management of chole-choledocholithiasis (24). Late biliary complications at 6 months were reported in two patients (3%) of the intraoperative ERCP group, in line with Elgeidie et al. (25) that reported complications at 30 days in only 1.4% of patients undergoing intraoperative ERCP. On the other hand, in our rendezvous cohort of patients, during 6 months of follow-up, we reported no late biliary complications, in line with Borzellino et al. (26), although La Barba et al. (27) showed an incidence of 5.5% at 5 years, with 4.5% of patients developing recurrent biliary stones.

Another advantage of the single-stage management compared with the two-stage approach is the reduction of the number of anesthetic procedures without increasing operating time. Baloyiannis et al. (2) reported that the single-stage approach is related with an approximately additional 30 min to be performed, and it saves more or less similar time in the endoscopic suite where ERCP is performed as a separate procedure. In our experience, the endoscopic time was significantly shorter in rendezvous groups with a median of 15 min compared with the median of 27 min in intraoperative ERCP. This limited additional time for the endoscopic procedure was strictly related to the great experience of the endoscopist and the excellent cooperation with the different surgeons.

Despite the advantages of single-stage technique, the greatest limitations are related to the logistics and organizational problems for a technique that requires the simultaneous presence of two teams (surgical and endoscopic) (2, 27, 28). Lella et al. (29), regarding the need for two teams and different equipment in the operating theatre, considered this technique more difficult to perform in an emergency setting. In our experience, we were able to manage this problem thanks to excellent cooperation between surgeons and endoscopists, even in the emergency setting. Moreover, most surgeons can perform ERCP, as stated by the American Society of Gastrointestinal Endoscopy (30).

The limitations of the study are its retrospective nature and single-center experience, which opens it to possible selection bias; a future prospective, multicenter randomized controlled study should be conducted.

## Conclusion

Laparoscopic rendezvous could be considered a preferable alternative to intraoperative ERCP for the treatment of patients with concomitant CBD stones and gallstones. This single-stage treatment avoids the main mechanism of iatrogenic pancreatic damage, leading to a lower incidence of postoperative pancreatitis. The lower overall morbidity reduces hospital stay and cost, with also reduced incidence of late biliary complications. However, the availability of this technique is still limited due to the expertise required and the cooperation needed between surgeons and endoscopists.

## Data Availability

The raw data supporting the conclusions of this article will be made available by the authors, without undue reservation.
